# Differential diagnosis of late onset psychotic symptoms. A case report.

**DOI:** 10.1192/j.eurpsy.2023.2256

**Published:** 2023-07-19

**Authors:** J. Sánchez Rodríguez, S. Puyal González, M. Paz Otero, E. Lozano Bori, A. García Recio

**Affiliations:** Psychiatry, Hospital Clínico San Carlos, Madrid, Spain

## Abstract

**Introduction:**

We present the case of a sixty-seven-year-old woman who is examined for the first time in the emergency room because of a nine-month clinical picture that involves psychotic symptoms. The patient exhibits persecutory delusions that started after she shared some private information on social media. These symptoms also entail emotional distress and behavioral disturbances. She has never experienced hallucinations, but she does present delusional interpretations of the environment. Her clinical history reveals abnormalities of premorbid personality, including paranoid and immature features.

**Objectives:**

(1) We will be carrying out a differential diagnosis of late onset psychotic symptoms. We will as well be exploring the concept of Paraphrenia and analyzing its differential features in order to stablish the most suitable diagnosis for the case.

(2) The relationship between abnormalities in premorbid personality and psychotic symptoms will be covered, reviewing the available literature on this matter, and relating it to the patient’s symptomatology.

**Methods:**

A review of the patient’s clinical history will be carried out, considering her biography, the testimony of her family and the complementary tests performed during the hospitalization period.

A bibliographic review of the available scientific literature will also be developed involving disorders that could explain psychotic symptoms in the elderly, as well as the term Paraphrenia, which describes specific features in a psychotic episode but is not included in the diagnostic manuals.

**Results:**

(1) The symptomatology that our patient exhibits may be considered atypical given the late stage of its onset and its specific features.

(2) The case could be explained under the category of Delusional Disorder. From a psychopathological point or view, it could also fit under de diagnosis of Paraphrenia as described by Ravindran et al.

(3) Pathological personality traits were assessed in premorbid personality which included paranoid and immature features.

**Conclusions:**

It could be useful to review the concept of the “paranoid spectrum” as proposed by some authors regarding some patient’s psychotic symptoms that don’t exactly fit any of the nowadays diagnostic categories. “Paraphrenia” could be understood as a distinct clinical entity for patients who show psychotic disorders but keep affective warmth and lack though deterioration and grossly disorganized behavior.

Most patients with late life paranoid psychoses have abnormal premorbid personalities, most usually of schizoid or paranoid type. There’s a decent amount of consensus in viewing the premorbid personality as having been abnormal as an early marker of impending psychosis.

Reformulating the way we approach diagnosis of psychotic symptoms of late onset could help us identify vulnerable patients on a premorbid stage and better classify and understand atypical entities.

**Disclosure of Interest:**

None Declared

